# State-of-the-Art Clinical Microbiology in South Korea: Current Trends and Future Prospects

**DOI:** 10.3390/microorganisms10010174

**Published:** 2022-01-14

**Authors:** Garima Sharma, Jin-Chul Kim

**Affiliations:** Department of Biomedical Science & Institute of Bioscience and Biotechnology, Kangwon National University, Chuncheon 24341, Korea

Researchers and clinicians have repeatedly explored the clinical aspects of microorganisms because the human body is inhabited by several different microbial species and their strains. The interaction between the microbial world and the human body is complicated and might lead to harmful or beneficial outcomes. On the one hand, some of the natural microbial inhabitants play an essential role in the metabolic activities of the human body [1]; alterations in the natural composition of the microbiota and various other microbial encounters influence the occurrence of infectious diseases of the gastrointestinal tract [2], urogenital tract [3], respiratory tract [4], central nervous system [5], etc. On the other hand, there has been an increase in implants or medical devices for various disease management in the past few decades. Bacterial colonization on these implants or medical devices has also severely threatened human health [6]. In the last few years, several new viral strains, such as ebola, Middle East respiratory syndrome coronavirus (MERS-CoV), and severe acute respiratory syndrome coronavirus-2 (SARS-CoV-2), etc., have been identified that have become a significant concern for public health worldwide [7]. Although, with the help of advancing technologies, the scientific community has carried out a tremendous amount of work exploring the cross-talk between the microbes and the host factors, there are still a lot of facts to uncover. Since different pathogeneses directly result from microbial infections, it is necessary to understand the relationship between the microbes and their associated health effects at the cellular and molecular levels. This would enable the development of novel antimicrobial agents. The development of antibiotic resistance by microbes is one of the biggest threats to human health [8], and thus, developing novel antimicrobial agents is the biggest challenge to the scientific community. 

In 2019, the world entered a COVID-19 pandemic caused by SARS-CoV-2. Since then, various lineages (i.e., variants of concern and variants of interest) of SARS-CoV-2 have also been reported due to escape mutations [9,10,11]. Among all the identified variants, B.1.1.529 (Omicron) is the most recently identified strain designated as a variant of concern by the World Health Organization (WHO) [12,13]. COVID-19 has been associated with various clinical manifestations, such as respiratory illness, dry cough, fever, dyspnea, secondary infections, sepsis, and organ failure, which have taken millions of lives to date [14]. It is plausible that the world may face more such threats in the future due to the constant mutations and evolutions in SARS-CoV-2 or other microbes. Indeed, the human immune system responds differently to different microbial encounters. Thus, to battle the severity of newly evolving microbes, it is crucial to uncover the microbial evolution and the dynamic interplay between them and host factors. This understanding might assist in developing antimicrobial drugs and vaccines for evolutionary-related microbial infections. 

Although the WHO now recommends multiple vaccines and repurposing drugs for managing the SARS-CoV-2 pandemic, their clinical efficiencies are still under trial [15]. Currently, the whole world is contributing its best efforts to develop various vaccines and drugs against COVID-19. In the attempt to combat COVID-19, 68 clinical studies related to drugs and vaccines against COVID-19 have been registered from South Korea in ClinicalTrials.gov (according to a search on 29 December 2021 using “COVID-19” as a keyword and “Korea, Republic of” as the country). Among them, 48 studies are either completed or are currently recruiting. Out of them, 39 studies are interventional and nine are observational, thus, signifying the efforts and current state of development of drugs and vaccines against COVID-19 in South Korea.

In view of the aforementioned, this Special Issue entitled “State-of-the-Art Clinical Microbiology in South Korea” focuses on recent advancements in the state of interactions between microbes and host factors associated with various pathogeneses. In addition, this issue also focuses on the state of the development of novel antimicrobial agents and vaccines to combat various microbial infections, including COVID-19. The contributions from leading authors from South Korea are intended to improve and expand our knowledge in the field of clinical microbiology. The planned papers are supposed to provide valuable and thought-provoking information that will give an insight into the microbe–host interactions to facilitate the development of novel antimicrobial agents and vaccines. We are honored to highlight the work of such expert groups of individuals from South Korea.
